# Bibliometric analysis of research articles on post-herpetic neuralgia published from 1991 to 2020

**DOI:** 10.1097/MD.0000000000032967

**Published:** 2023-02-10

**Authors:** Jeongsoo Kim, Hyeon Cheun, Jeong Jeong, Ho-Jin Lee

**Affiliations:** a Department of Anesthesiology and Pain Medicine, Seoul National University Hospital, Seoul, Republic of Korea; b Department of Anesthesiology and Pain Medicine, SMG-SNU Boramae Medical Center, Seoul, Republic of Korea; c Department of Anesthesiology and Pain Medicine, Seoul National University College of Medicine, Seoul, Republic of Korea.

**Keywords:** bibliometrics, nerve block, neuralgia, pain clinics, postherpetic

## Abstract

Post-herpetic neuralgia (PHN) is one of the most painful diseases, which has made it a major concern for pain physicians. We aimed to quantitatively analyze the research outputs of studies on PHN published over the past 30 years using bibliometric analysis. We also aimed to analyze the research outputs of studies on interventional treatments for PHN and evaluate the academic achievements of Korean pain physicians. Bibliometric analysis was performed by searching the Web of Science database for PHN-related articles published between 1991 and 2020. Publication number, year, source, country, institution, and citation-related information were retrieved from the database. We also quantitatively analyzed publications related to interventional treatments for PHN. A total of 3285 publications were extracted from the database; 101 (3.1%) of the articles were published by South Korean authors, making South Korea the 11th in the order of countries that published the most articles. There were 185 articles on the effects of interventional treatments for PHN. South Korean authors published 30 (16.2%) articles out of these, making South Korea the 3rd in the order of countries that published the most articles on the effects of interventional treatment for PHN. Our results showed an increasing trend in the number of PHN-related publications and the academic achievements of Korean pain physicians in this field over the past 3 decades. However, the proportion of studies on interventional treatments is relatively small. Korean pain physicians need to establish academic evidence on interventional treatment to expand their role in this field and improve the outcomes of PHN patients.

## 1. Introduction

Post-herpetic neuralgia (PHN) is one of the most painful types of neuropathy.^[1]^ It is the most common complication of herpes zoster (HZ) and is defined as persistent HZ-related pain 3 months after the onset of the HZ rash.^[2]^ The incidence of PHN in South Korea is reported to be 2.5 per 1000 person-years, which is relatively high compared to that of other countries.^[3]^ According to the Healthcare Big Data Hub of the Korean Health Insurance Review & Assessment Service of South Korea,^[4]^ the number of patients with PHN has increased by about 73% from 90,706 in 2010 to 157,141 in 2019, and its prevalence is expected to increase as the population ages. Considering its high prevalence and significant impact on the quality of life,^[5]^ the prevention and treatment of PHN is an important task for pain physicians.

Bibliometric analysis is a useful method for quantitative and qualitative identification of research trends in specific fields, which can provide valuable information about future research.^[6]^ Researchers can easily identify research topic trends through bibliometric analysis, which analyzes articles or books in specific fields from the past to the present. Bibliometric analysis also allows researchers to understand the research composition of particular fields by displaying the countries, institutions, and authors that have been actively involved. The information obtained through bibliometric analysis in specific fields can reveal the most interesting scientific publications or topics up to the present time, and, on the other hand, it may reveal the need for additional studies on underrepresented topics. Bibliometric analysis has recently been used to identify research trends in various fields of medicine,^[7–11]^ including pain medicine.^[12,13]^ However, despite the surge in PHN-related research, to the best of our knowledge, there has been no bibliometric analysis-based report in this field to date.

Therefore, we aimed to quantitatively analyze the research outputs of PHN-related studies published globally and in South Korea over the past 30 years. We also aimed to quantitatively analyze the research outputs of PHN- related interventional treatments commonly conducted by pain physicians.

## 2. Material and Methods

Since we only used public databases, the ethical review of this study was exempted by the institutional review board of Seoul National University Hospital. We retrieved PHN-related articles published between 1991 and 2020 from the Web of Science Core Collection (WOS) database, which has most commonly been used in bibliometric analyses.^[14]^ The search terms used are as follows: Theme = “postherpetic neuralgia*” OR “postherpetic pain*.” The themes included title, abstract, author keywords, and keywords plus^®^.^[15]^ Only peer-reviewed articles and reviews written in English were included in the analysis. Details such as the original country of research, institutes, journals, research category, and citation information were retrieved from the WOS database. We also retrieved the 10 most cited articles by conducting a search using the following search terms: Title = “postherpetic neuralgia*” OR “postherpetic pain*,” and the period of citation was defined as until 2020. Since the studies found with the above search terms included articles on various types of neuropathic pain, including PHN, we used title instead of theme to retrieve articles directly related to PHN.

We used the VOSviewer software (Version 1.6.16, Leiden University, Leiden, Netherlands) for mapping and clustering of keywords (access date: March 8, 2021).^[16]^ The software estimates the “similarity” (affinity) of terms based on the number of their co-occurrences in the title or abstract of the same publication, using the “association strength” measure proposed by Van Eck and Waltman.^[17]^ The larger the number of publications in which 2 terms co-occur, the stronger the terms are considered to be related to each other. Therefore, terms that often co-occur in the same publications are located close to each other in a “term map,” whereas terms that are less strongly related (low co-occurrence) are located further away from each other. Graphically, each term is represented by a circle, where the diameter and size of its label indicate the number of occurrences of the corresponding term in the title or abstract of publications. VOSviewer can classify related keywords into disparate clusters using a weighted and parameterized variant of modularity-based clustering.^[18]^ Additionally, it can classify keywords into a disparate cluster according to the average publication year. The average publication year is the average year of the publications in which a keyword occurs. This measure can be used to evaluate the relative novelty of a keyword.

Two board-certified pain physicians (J Kim and H-J Lee) evaluated the number of publications on the effect of PHN-related interventional treatments and assessed the types of interventional treatments used in the studies by reviewing the titles and abstracts of the articles. Interventions not implemented by our department, such as ablative surgery, computed tomography-guided intervention, transcranial magnetic stimulation, and ozone injection, were excluded. Subcutaneous or transcutaneous stimulation and subcutaneous injection of therapeutics were excluded as well.

We conducted only descriptive statistical analysis using the MedCalc Statistical Software version 18.6 (MedCalc Software bvba, Ostend, Belgium). Categorical data are described as percentages.

## 3. Results

**Figure 1.**
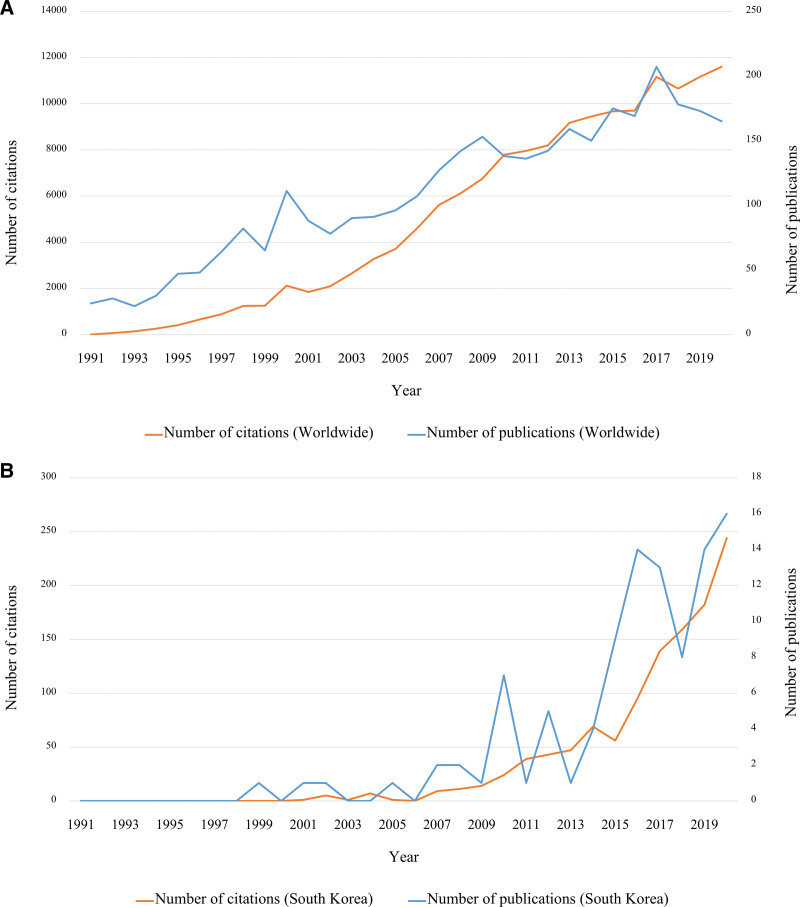
The number of postherpetic neuralgia-related articles and citations published (A) worldwide and (B) in South Korea over the past 30 years.

A total of 3285 PHN-related articles have been published worldwide over the past 30 years. The number of articles published showed an increasing trend over the years (Fig. 1A). The total number of citations, average citations per item, and h-index of PHN-related articles published worldwide over the past 30 years were 152,251; 46.35; and 171, respectively. A total of 101 (3.1%) articles have been published in South Korea over the past 30 years. The number of articles published in South Korea also showed an increasing trend over the years (Fig. 1B). The total number of citations, average citations per item, and h-index of PHN-related articles published in South Korea over the past 30 years were 1190; 11.78; and 20, respectively. The 10 most cited articles directly related to PHN are shown in Table 1.^[19–28]^

**Table 1:** The 10 most cited articles concerning postherpetic neuralgia.

Article	Author	Journal	Publication yr	Total citation	Average citations per yr
A vaccine to prevent herpes zoster and postherpetic neuralgia in older adults^[19]^	Oxman MN et al	*New England Journal of Medicine*	2005	1437	47.9
Gabapentin for the treatment of postherpetic neuralgia: a randomized controlled trial^[20]^	Rowbotham M et al	*Journal of the American Medical Association*	1998	1005	33.5
Pregabalin for the treatment of postherpetic neuralgia: a randomized, placebo-controlled trial^[21]^	Dworkin RH et al	*Neurology*	2003	537	17.9
Efficacy of oxycodone in neuropathic pain: a randomized trial in postherpetic neuralgia^[22]^	Watson CP et al	*Neurology*	1998	436	14.5
Gabapentin in postherpetic neuralgia: a randomized, double blind, placebo controlled study^[23]^	Rice ASC et al	*Pain*	2001	426	14.2
Postherpetic neuralgia pathogenesis, treatment, and prevention^[24]^	Kost RG et al	*New England Journal of Medicine*	1996	412	13.7
Both intravenous lidocaine and morphine reduce the pain of postherpetic neuralgia^[25]^	Rowtbotham MC et al	*Neurology*	1991	391	13
Epidemiology and impact on quality of life of postherpetic neuralgia and painful diabetic neuropathy^[26]^	Schmader KE.	*Clinical Journal of Pain*	2002	377	12.6
Opioids versus antidepressants in postherpetic neuralgia: a randomized, placebo-controlled trial^[27]^	Raja SN et al	*Neurology*	2002	366	12.2
Relief of postherpetic neuralgia with the n-methyl-d-aspartic acid receptor antagonist ketamine: a double-blind, cross-over comparison with morphine and placebo^[28]^	Eide PK et al	*Pain*	1994	364	12.1

**Figure 2.**
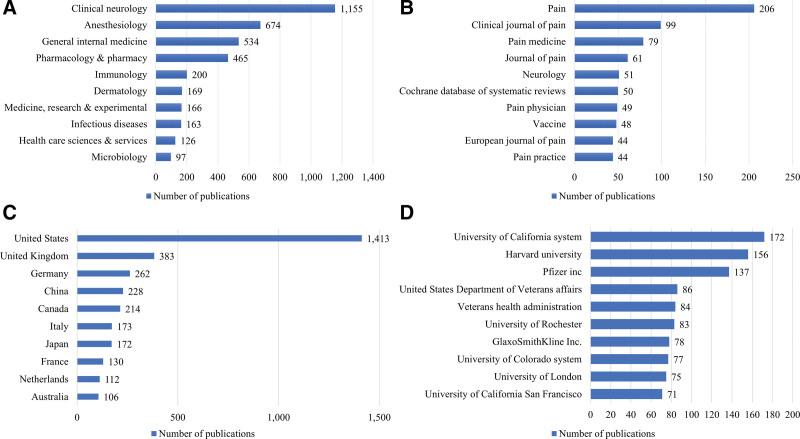
The number of postherpetic neuralgia-related articles published in the top 10 (A) research categories, (B) journals, (C) countries, and (D) institutions.

Figure 2 shows the number of PHN-related articles published in the top 10 research categories, journals, countries, and institutions. The most common research category was clinical neurology (n = 1017; 31.0 %), followed by anesthesiology (n = 674, 20.5 %). In South Korea, the most common research category was clinical neurology (n = 22, 21.8 %), followed by general internal medicine (n = 21, 20.8 %). The journal that published the highest number of PHN-related articles was *Pain* (n = 206, 6.3 %), which had an impact factor (IF) of 5.438 in 2019, followed by *Clinical Journal of Pain* (n = 99, 3.0 %), which had an IF of 2.893 in 2019. The journal that published the highest number of PHN-related articles in South Korea was *Medicine* (n = 9), which had an IF of 1.552 in 2019, followed by *Korean Journal of Pain* (n = 8), which had an IF of 1.431 in 2019. The country that published the most articles related to PHN was the US (n = 1413, 43.0 %) followed by the UK (n = 383, 11.7 %); South Korea (n = 101, 3.1 %) ranked 11th in this order. The institution that published the most articles related to PHN was the University of California system (n = 172, 5.2 %), with an h-index of 59; followed by Harvard University (n = 156, 4.7 %), with an h-index of 56; and Pfizer Inc., with an h-index of 50. In South Korea, the institution that published the most articles related to PHN was the Catholic university (n = 27), with an h-index of 13; followed by Korea University (n = 23), with an h-index of 9; and Seoul National University (n = 21), with an h-index of 10.

**Figure 3.**
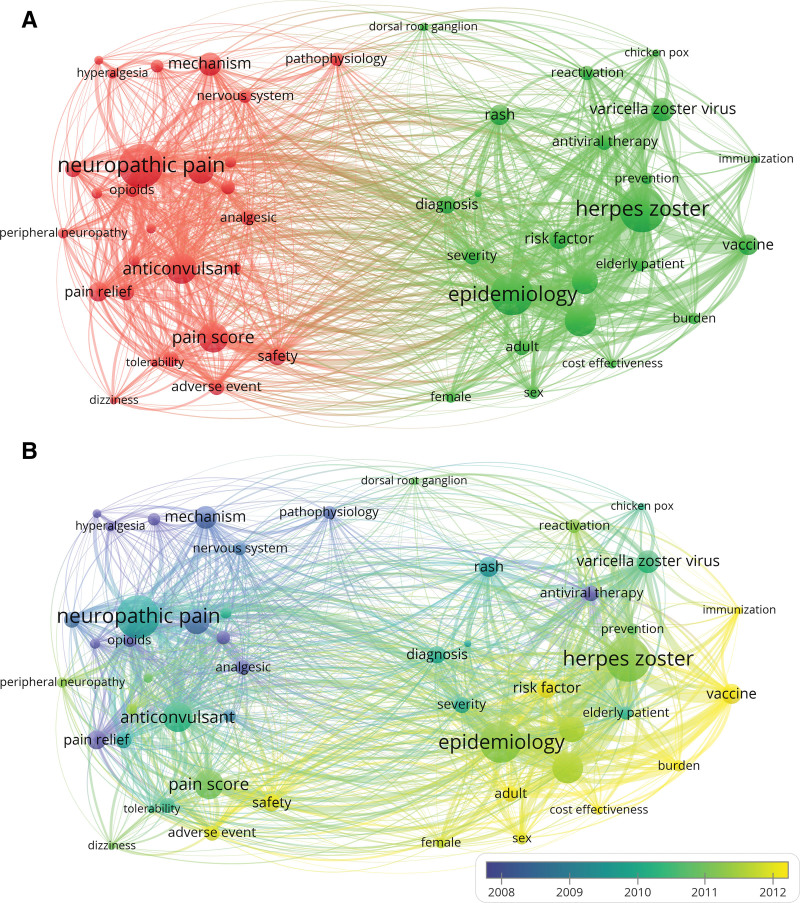
Network visualization map of (A) co-occurrence of keywords and (B) time when a keyword appears in postherpetic neuralgia-related studies. Keywords in yellow appeared later than those in blue.

A total of 61 keywords were extracted from the titles and abstracts of the articles. The most frequent keyword was “herpes zoster” (n = 1077), followed by “neuropathic pain” (n = 1043) and “epidemiology” (n = 991). The keywords were classified into 2 clusters. Cluster 1 was mainly about symptoms, treatment, and mechanisms (Fig. 3A, red), whereas Cluster 2 was mainly related to epidemiology and prevention (Fig. 3A, green). Regarding the differences between the average publication years of keywords, the keywords in Cluster 2 appeared in more recent years than those in Cluster 2 (Fig. 3B). “Vaccine” had the latest average publication year, which was 2013.26, followed by “burden,” which had an average publication year of 2013.18.

**Figure 4.**
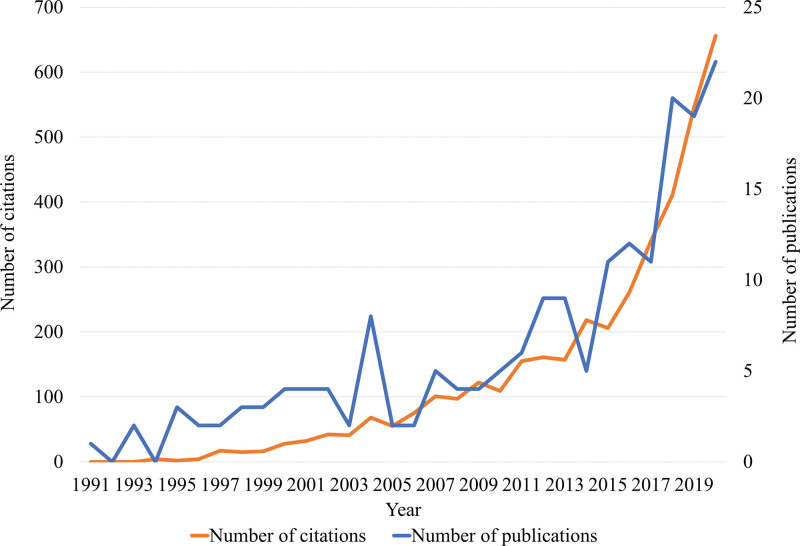
The number of publications and citations on interventional treatment-related research in the postherpetic neuralgia-related articles.

**Figure 5.**
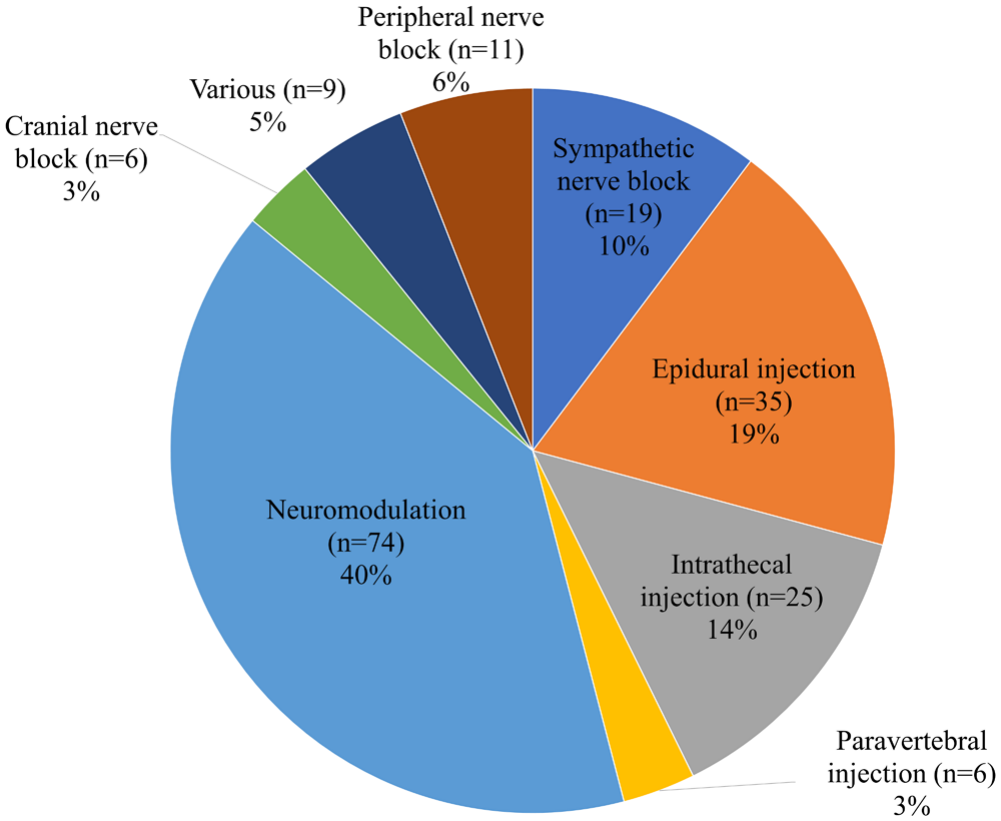
(B) The types of interventions utilized in interventional treatment-related researches.

**Figure 6.**
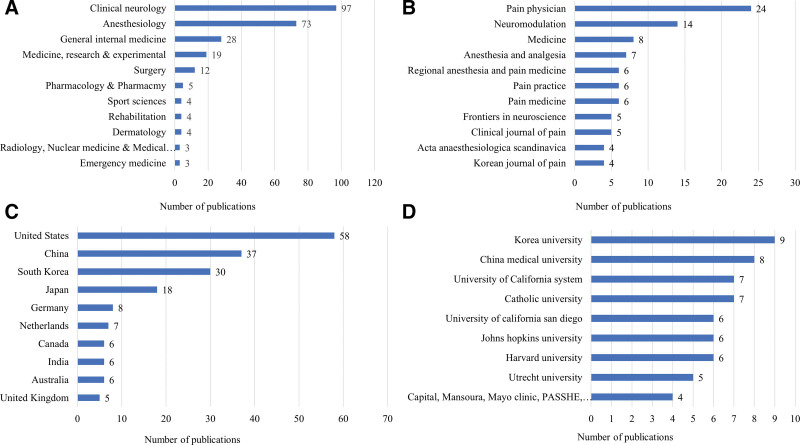
The number of interventional treatment-related articles published in the top 10 (A) research categories, (B) journals, (C) countries, and (D) institutions. PASSHE, Pennsylvania State System of Higher Education.

There were 185 articles on the effects of interventional treatments. The trends in the volume and citation of interventional treatment-related research published over the past 30 years are shown in Figure 4. The total number of citations, average citations per item, and h-index were 4035; 21.93; and 32, respectively. Articles related to neuromodulation (n = 74, 40.0 %) were the most common, followed by those on epidural injections (n = 35, 18.9 %) (Fig. 5). The top 10 research categories, journals, countries, and institutions are presented in Figure 6; South Korea (n = 30, 16.2 %) ranked third in this order globally. The total number of citations, average citations per item, and h-index of the interventional treatments described in PHN-related articles published in South Korea were 265, 8.83, and 10, respectively.

## 4. Discussion

Our study revealed an increasing trend in the number of PHN-related publications over the past 3 decades, which indicates that this painful neuropathy has consistently attracted researchers’ attention. We also found that Korean pain physicians have made a significant contribution to PHN-related research, especially intervention-related research. However, intervention-related studies have been a relatively small proportion in this field. This study provides valuable information regarding future studies by pain physicians.

According to our results, the interest in PHN-related research has shifted from the symptoms and pharmacological treatment of PHN to identifying its risk factors and prevention over the past three decades. Considering the high prevalence and significant economic burden of HZ,^[29,30]^ its prevention and the subsequent occurrence of PHN would be the most important task in terms of cost-effectiveness. The introduction of the live attenuated zoster vaccine was a cornerstone in the prevention of HZ and has effectively reduced its incidence and that of PHN.^[31]^ Further, the recently developed non-live recombinant zoster vaccine (Shingrix^®^), which has not yet been introduced in Korea, has shown promising results and is expected to reduce the incidence of HZ and PHN more effectively.^[32]^ In our analysis, we found that “vaccine” is the most recent topic of PHN-related studies, and that further studies on the prevention of HZ and subsequent PHN using vaccines are expected to increase. Additionally, the mechanisms of the progression from HZ to PHN are still unclear. Thus, further research is required to clarify the mechanisms behind the disease progression to prevent and treat PHN effectively.^[33]^

Another notable result of our study is the relatively small proportion of interventional treatment-related studies in this field. The investigation of interventional treatments for PHN was not planned at the early stage of the study; however, we conducted the investigation after the keyword network analysis confirmed a low frequency of keywords related to interventional treatments. The average citation per item for interventional treatment-related research was only about half of that for all the articles. Interventional treatments including neuraxial and sympathetic blockades can reduce HZ-related pain and prevent the progression to PHN.^[2]^ However, despite the widespread use of interventional treatments for the treatment of PHN in clinical practice,^[34]^ the evidence backing its usage is not yet solid. According to the recommendations published by the Neuropathic Pain Special Interest Group of the International Association for the Study of Pain, only epidural injections are recommended for HZ-related pain. However, the degree of recommendation for epidural injections is low, whereas the use of sympathetic blocks for PHN is not recommended at all.^[35]^ In a recently published systematic review, the degree of recommendation for various interventions except intrathecal steroid injection was still not high.^[36]^ The main reason for the low degrees of these recommendations would be insufficient evidence, especially the paucity of well-controlled randomized controlled studies, rather than the low effectiveness of the interventions.^[36]^ Although the preventive effect of nerve block for PHN has been reported in several studies,^[37–39]^ the negative outcomes of its preventive effects have been reported in a well-designed randomized controlled trial.^[40]^

Further research on the effects of interventional treatments, especially neuromodulatory procedures, and their appropriate timing in the treatment of patients with PHN that is refractory to conservative treatments are also required. Although anticonvulsants, antidepressants, and opioids have been proven to be effective in the treatment of PHN, some patients do not respond to these medications. Despite the insufficient evidence of the efficacy of neuromodulatory procedures, we expect that neuromodulatory procedures will be effective in improving the prognoses of patients with refractory PHN.^[36,41,42]^

Eventually, these efforts will expand the role of pain physicians in the treatment of HZ and subsequent PHN. Since HZ is a viral disease and its initial symptoms are mainly skin symptoms such as rashes, most patients with HZ visit other departments before visiting a pain physician.^[34]^ However, unlike other physicians, pain physicians can provide interventional treatment as well as medication. Furthermore, the effectiveness of an intervention such as a nerve block has been reported to be associated with its timing in patients with PHN.^[38]^ Therefore, visiting the pain clinic as early as possible will improve the outcomes of patients with HZ. In a retrospective study conducted in Korea, it was reported that patients with PHN who had shorter first visits to the pain clinic visit showed better outcomes.^[43]^

Our study had several limitations. First, we retrieved publications from the WOS database only; thus, the generalizability of the results may be dependent on the reach of the database.^[14]^ However, since the WOS database only indexes journals in the Science Citation Index Expanded and Emerging Science Citation Index that are generally recognized by researchers.^[44]^ Second, since we found it difficult to identify the contents of publications written in other languages, we only included publications written in English. Several academic achievements written in other languages may not have been included. Third, since we evaluated the relative importance and trend of the research topic only through the co-occurrence of the terms in the titles, abstracts, and keywords, we could not identify the details of each publication.^[45]^ Finally, we did not consider the relative contributions of each author or institution in collaboration studies that included different institutions or authors from different countries. This may have caused a bias in the distribution of institutions and countries.

In conclusion, we identified the academic evolution of PHN-related research over the past three decades using bibliometric analysis. Our results showed that researchers’ interest in PHN has continued to increase. However, we also suggested a future direction for them regarding PHN-related research. Pain physicians should establish academic evidence of interventional treatments to expand their role in this field and improve the outcomes of patients with HZ and PHN.

## Author contributions

**Conceptualization:** Jeongsoo Kim, Ho-Jin Lee.

**Data curation:** Hyeon Cheun, Jeong Jeong.

**Investigation:** Hyeon Cheun, Jeong Jeong.

**Supervision:** Ho-Jin Lee.

**Writing – original draft:** Jeongsoo Kim.

**Writing – review & editing:** Ho-Jin Lee.
